# Position-Dependent Hemodynamics of Popliteal Artery Stent Grafts: The POPFLEX Study

**DOI:** 10.3390/mps9050133

**Published:** 2026-09-17

**Authors:** Christos Chronis, Konstantinos Tzirakis, Nikolaos Kontopodis, Nikolaos Galanakis, Elias Kehagias, Christos V. Ioannou

**Affiliations:** 1 Vascular Surgery Department, Medical School, University of Crete, 71110 Heraklion, Greece; medp2012129@med.uoc.gr (C.C.); nkontopodis@uoc.gr (N.K.); 2 Department of Mechanical Engineering, School of Engineering, Hellenic Mediterranean University, 71004 Heraklion, Greece; ktzirakis@hmu.gr; 3 Interventional Radiology Unit, Medical School, University of Crete, 71003 Heraklion, Greece; ngalanakis@uoc.gr (N.G.); eliaskmd@yahoo.gr (E.K.)

**Keywords:** popliteal artery aneurysm, stent thrombosis, computational fluid dynamics, medical image segmentation, three-dimensional reconstruction, mesh optimization, hemodynamics, open-source software, CT angiography

## Abstract

**Background:** Endovascular exclusion of popliteal artery aneurysms (PAAs) with long covered stent-grafts is valuable for patients unfit for open surgery but is undermined by late stent thrombosis, even in fully scaffolded segments without kinking, a failure best clarified by computational fluid dynamics (CFD), which requires anatomically accurate 3D models—difficult to obtain in the mobile knee. **Methods:** In this prospective, single-centre study, consecutive patients undergoing endovascular PAA repair over 24 months (≥20 patients) underwent CT angiography in three knee positions (full extension, 45° and 90° flexion). Every treated limb was included, with limb as the unit of analysis. Images undergo a geometric pipeline: segmentation in 3D Slicer, then refinement in MeshLab, Meshmixer and VMTK. Meshes are converted to hexahedral volumes (ANSA Cadense) and simulated under pulsatile flow (ANSYS Fluent). Primary endpoints are time-averaged wall shear stress (TAWSS), oscillatory shear index (OSI) and relative residence time (RRT) over the stented segment, plus the surface proportion meeting thrombogenic criteria; positions are compared via linear mixed-effects models with limb nested within patient. **Results:** The protocol reproducibly yields watertight, artefact-free models preserving physiological morphology and supporting TAWSS, OSI and RRT quantification across knee positions. **Conclusions:** This protocol standardizes generation of CFD-ready popliteal geometries, enabling positional analysis of stent thrombosis.

## 1. Introduction

Popliteal artery aneurysms (PAAs) are the most frequently encountered peripheral aneurysms and pose a severe threat to limb viability [1]. Although open surgical repair remains the traditional gold standard, endovascular exclusion with long covered stent-grafts has become a crucial alternative for patients unfit for open surgery, typically those of advanced age or with severe cardiopulmonary comorbidity such as coronary artery disease, congestive heart failure or advanced chronic obstructive pulmonary disease [2].

In this highly mobile region, however, endovascular repair is limited by late stent thrombosis, which frequently precipitates acute limb ischaemia and carries a high risk of major amputation if immediate revascularisation is not achieved [3]. Notably, thrombosis often occurs in the absence of acute localized mechanical kinking, and in many such cases the implanted stents span the knee joint continuously. Despite this complete scaffolding, the mechanisms of failure remain poorly understood, and the fluid dynamics of extended stented popliteal conduits under varying knee positions have not been investigated.

To address this gap, we designed a study in which CTA of the treated popliteal artery is acquired in full extension and in 45° and 90° of knee flexion [4,5,6,7,8,9] and translated into position-specific hemodynamic models by computational fluid dynamics (CFD), in order to test whether positional flow disturbances—altered wall shear stress or regions of near-wall stasis—account for the thrombotic events observed in vivo.

The validity of such simulations depends entirely on the geometric accuracy of the underlying three-dimensional models, and generating watertight surface meshes from multi-positional DICOM data of the flexed knee is technically demanding: threshold-based segmentation yields intersecting faces, artefactual spikes and disconnected micro-fragments arising from imaging noise and from the proximity of osseous structures during flexion. Commercial packages automate much of this correction but their cost limits accessibility, whereas open-source tools are powerful yet lack standardized methodologies, so that results are frequently irreproducible or geometrically distorted by aggressive, volume-shrinking smoothing. This paper therefore reports a standardized, reproducible pipeline based on 3D Slicer, MeshLab, Autodesk Meshmixer and VMTK that extracts vascular geometries from multi-positional DICOM datasets, systematically eliminates artefacts, and optimizes the final mesh while preserving the true physiological morphology required for CFD analysis.

## 2. Experimental Design

### 2.1. Study Design, Population and Recruitment

POPFLEX is a prospective, single-centre observational cohort study conducted at the University Hospital of Heraklion. Recruitment is consecutive: every patient undergoing endovascular exclusion of a popliteal artery aneurysm with a long covered stent-graft at the participating centre during a 24-month enrolment window is screened for eligibility and, if eligible and consenting, enrolled. No convenience sampling is performed. On the basis of an institutional caseload of approximately 15 such procedures per year, a minimum of 20 patients are anticipated over the recruitment period.

Every treated limb is included, and in patients with bilateral disease treated within the study window both limbs are enrolled. The limb rather than the patient is therefore the unit of analysis, and the statistical model accounts for the clustering of limbs within patients (Section 2.4).

Eligibility criteria are those applied in routine clinical practice at the participating centre, in accordance with current European Society for Vascular Surgery guidance. Inclusion criteria are: (i) age ≥ 18 years; (ii) a popliteal artery aneurysm meeting the accepted clinical indication for repair; (iii) treatment by endovascular exclusion with a long covered stent-graft spanning the knee joint; (iv) ability to tolerate CTA acquisition in full extension and in 45° and 90° of knee flexion; and (v) written informed consent. Exclusion criteria are: (i) treatment by open surgical repair; (ii) any contraindication to iodinated contrast medium, including renal impairment below the institutional threshold; (iii) orthopedic or musculoskeletal limitation preventing 90° of knee flexion; (iv) presentation with acute limb ischaemia requiring emergency intervention that precludes protocol-compliant imaging; and (v) inability to provide informed consent.

Because inclusion follows the clinical indication for treatment, no patient is exposed to an additional intervention for study purposes. The only study-specific procedure is the acquisition of the two flexed-knee CTA series in addition to the extension series obtained for procedural planning and surveillance. The study is approved by the Institutional Ethics Committee, and all participants provided written informed consent.

### 2.2. Materials and Software

The geometry reconstruction stages of this protocol (Parts A and B) rely entirely on validated open-source or freely available software, ensuring that the most operator-dependent portion of the workflow is globally accessible and reproducible. The subsequent volume meshing and simulation stages (Part C) use commercial packages.

The workflow requires a standard workstation with adequate processing power to handle large multi-slice Computed Tomography (CT) datasets and dense mesh calculations.

**3D Slicer (Version 5.10.0):** An open-source software platform for medical image informatics, image processing, and three-dimensional visualization [10]. In this protocol, it is utilized for the primary processing of DICOM data, threshold-based segmentation, and initial artefact isolation.

**MeshLab (Version 2025.07):** An open-source, advanced 3D mesh processing system. It is employed in this workflow for its robust algorithms regarding isotropic remeshing, topological cleaning, and volume-preserving smoothing (Taubin) [11].

**Autodesk Meshmixer (Version 3.5):** A free software dedicated to working with triangle meshes. It is uniquely suited for this protocol due to its localized, brush-based sculpting tools, allowing for the manual removal of non-physiological surface defects (spikes and bumps) without affecting the global geometry.

**VMTK—Vascular Modelling Toolkit (Version 1.4):** An open-source collection of libraries and command-line tools for the geometric analysis of tubular structures in medical images [12]. It is used here for volume-preserving surface smoothing, for interactive clipping of the inlet and outlet planes, and for the centreline-based calibre measurements that verify the smoothing correction in Step 9.

**ANSA Cadense 24.1.7:** A commercial CAE pre-processing package [13], used for planar capping of the clipped boundaries, property identifier assignment, and generation of the hexahedral volume mesh with a near-wall O-Grid.

**ANSYS Fluent (Edition 19.2):** A commercial finite-volume CFD solver [14], used for the transient pulsatile simulations and, through user-defined functions, for the computation of TAWSS, OSI and RRT.

### 2.3. Outcome Measures and Definition of Thrombogenic Thresholds

Hemodynamic indices are evaluated over the luminal surface of the stented segment, delimited by the proximal and distal stent-graft edges, and are computed from the final (fifth) simulated cardiac cycle.

The primary outcome measures are: (i) the cycle-averaged time-averaged wall shear stress (TAWSS), oscillatory shear index (OSI) and relative residence time (RRT), each expressed as the spatial mean over the stented luminal surface; and (ii) the thrombogenic surface-area fraction, defined as the percentage of the stented luminal surface simultaneously exposed to TAWSS ≤ 0.4 Pa, OSI ≥ 0.3 and RRT ≥ 10 Pa^−1^. The fraction satisfying each of the three criteria individually is also reported.

The secondary outcome measures are the same indices evaluated at peak systole and at late diastole, together with descriptors of flow separation and recirculation, namely the number, axial location and volume of recirculation zones.

Definition of thrombogenic thresholds. Thresholds are pre-specified from established literature rather than defined post hoc. Wall shear stress in healthy arteries lies approximately between 1 and 7 Pa, and sustained values of ≤0.4 Pa are associated with endothelial dysfunction and a pro-thrombotic endothelial phenotype [15,16,17]. An OSI ≥ 0.3 identifies surfaces subject to substantial cycle-to-cycle reversal of the shear vector, and RRT, which combines shear magnitude and directionality into a single measure of near-wall residence, is applied with a threshold of 10 Pa^−1^.

### 2.4. Statistical Analysis Plan

**Unit of analysis and data structure.** The unit of analysis is the treated limb. Each limb contributes three geometries and therefore three sets of hemodynamic indices, one per knee position. Surface nodes are not treated as independent observations: nodal fields are reduced to the limb-level summary measures defined in Section 2.3 before any inferential analysis, so that the number of observations equals the number of limb–position combinations.

**Primary analysis.** Because the three positions constitute a within-limb repeated-measures factor, each outcome is analyzed with a linear mixed-effects model containing knee position as a three-level categorical fixed effect and a random intercept for limb nested within patient. This specification accounts simultaneously for the repeated measurement of the same limb across positions and for the clustering of limbs within patients in bilateral cases. Full extension serves as the reference level. Positional effects are reported as adjusted mean differences with 95% confidence intervals, derived from estimated marginal means, and the three pairwise comparisons between positions are corrected by the Holm method.

**Assumptions and pre-specified alternatives.** Model assumptions are examined by inspection of residual and random-effect quantile–quantile plots and of residual-versus-fitted plots. Right-skewed outcomes, in particular RRT, are analyzed after natural-logarithmic transformation, and surface-area fractions after logit transformation or, equivalently, by beta regression with the same random-effects structure. Should the assumptions of the mixed model remain untenable, the pre-specified non-parametric alternative is the Friedman test across the three positions, with Wilcoxon signed-rank post hoc tests and Holm correction, restricted to limbs with complete data.

**Effect sizes.** Effect sizes are expressed in three complementary ways: as adjusted mean differences with 95% confidence intervals in the original units of each index; as standardized within-limb differences (Cohen’s d_2_) for each pairwise positional contrast; and as marginal and conditional R^2^ for each mixed model. The intraclass correlation coefficient of the random intercepts is reported to quantify the magnitude of the within-limb dependence.

**Significance level.** All tests are two-sided with α = 0.05. TAWSS, OSI and RRT are pre-specified as co-primary indices; the family of pairwise comparisons within each index is protected by Holm correction, whereas no further adjustment is applied across the three indices, and this is stated as a limitation of the analysis.

**Sample size.** This is a methodological protocol rather than a hypothesis-testing trial, and recruitment is therefore determined by the institutional caseload over the 24-month enrolment window rather than by a formal power calculation. The anticipated minimum of 20 patients corresponds to approximately 20 to 25 limbs and 60 to 75 simulations. For orientation, a within-limb paired comparison of 20 limbs provides 80% power at α = 0.05 (two-sided) to detect a standardized within-limb difference of d_2_ ≈ 0.66, which is compatible with the magnitude of positional geometric and hemodynamic change [5,6,7,8,9].

## 3. Procedure

**Figure 1:**
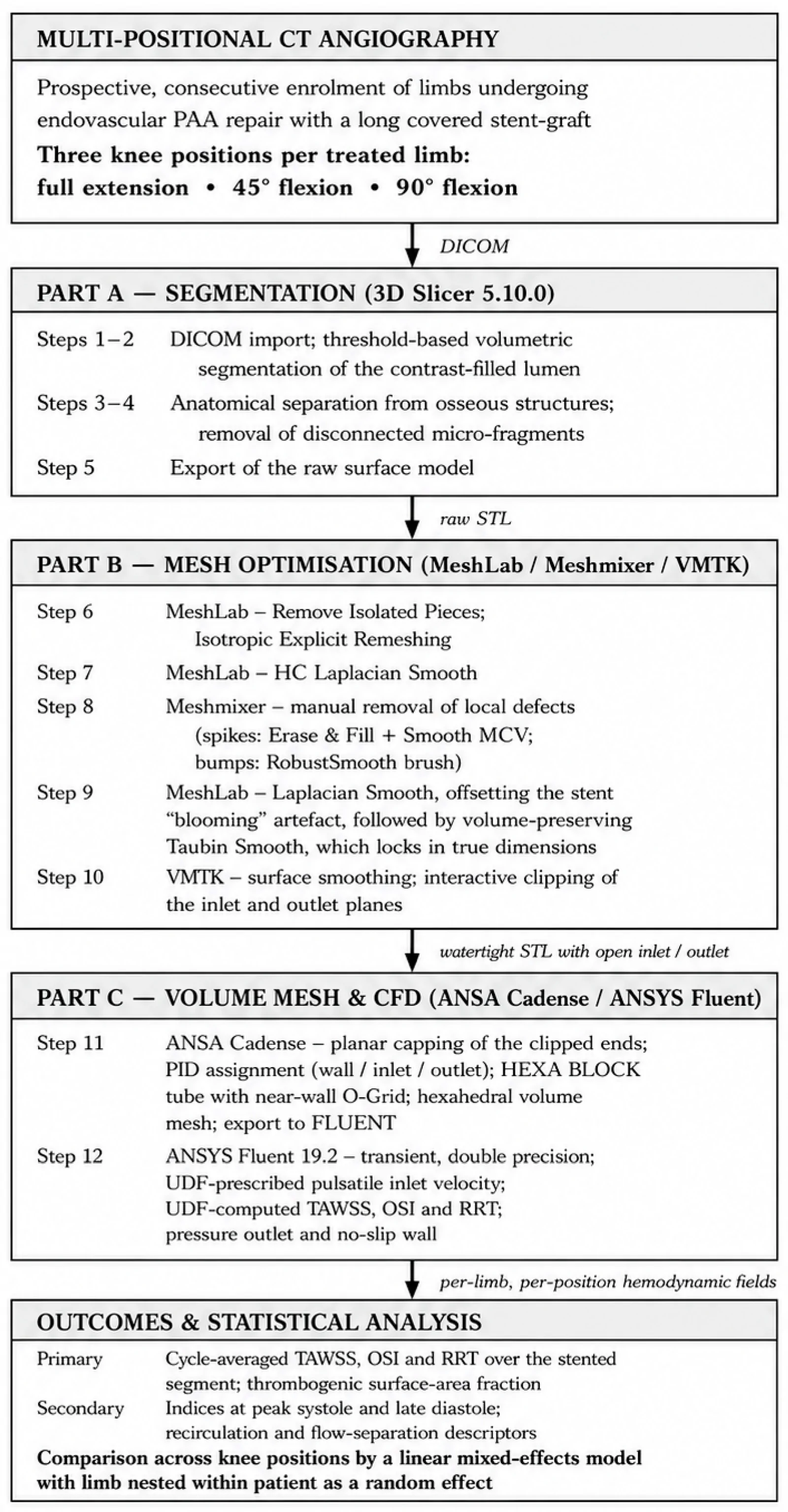
Graphical overview of the complete pipeline.

The workflow is divided into three stages: (A) volumetric segmentation and extraction from DICOM data, (B) topological refinement and optimization of the resulting surface model, and (C) volume mesh generation and blood flow simulation. Figure 1 provides a graphical overview of the complete pipeline, the sections that follow describe each step in the detail required for independent reproduction.

### 3.1. Part A: Model Generation and Anatomical Separation (3D Slicer)

**Step 1: Data Import and Region of Interest (ROI) Definition.** The multi-positional patient DICOM dataset is loaded into 3D Slicer. The Window Level parameters are adjusted to optimize the visual contrast of the peripheral vasculature. The Crop Volume module is used to define a strict bounding box (ROI) exclusively around the popliteal artery and adjacent vessels of interest. This step is critical for reducing computational load and isolating the target anatomy. The Apply button is then clicked, and the output volume is renamed appropriately to designate laterality (e.g., “Rt” or “Lt”).

**Step 2: Threshold-Based Segmentation.** Within the Segment Editor module, a new segment is added and the Threshold tool is selected. A baseline lower threshold of 200 HU is generally recommended to capture the contrast-enhanced blood pool. However, this value must be dynamically adjusted within a specific range (typically 180–250 HU) depending on the exact contrast enhancement phase and the specific CT scanner calibration used. Apply is then clicked to generate the initial 3D volumetric representation of the vessel lumen.

**Step 3: Anatomical Cleaning and Isolation.** To eliminate scattered imaging noise (“islands”), the Islands tool is selected and the Keep Selected Island function is applied. Clicking directly on the main body of the popliteal artery automatically deletes all disconnected extraneous voxels. Due to the proximity of the popliteal artery to the knee joint during flexion, artifactual connections with adjacent osseous structures often occur. The Scissors tool (set to Erase Inside) is used to manually sever these unwanted bony connections. The Islands tool (Keep Selected Island) is then reapplied to discard the newly detached bone fragments.

**Step 4: Truncation for CFD Boundary Conditions.** To ensure identical velocity profiles during CFD simulations, the inlet and outlet boundaries must be perfectly planar. The camera is aligned perfectly perpendicular to the vessel using the Snap View feature. In the Segment Editor, the Scissors tool is configured to a Rectangle shape, and orthogonal cuts are performed at the proximal inlet and distal outlet of the popliteal segment. The Islands tool (Keep Selected Island) is used one final time to isolate the finalized region of interest.

**Step 5: Initial Surface Smoothing and Export.** The Smoothing tool within the Segment Editor is applied using the Median method with a Kernel Size of 1.50 mm. This mitigates the “staircase” effect inherent to the slice thickness of CT data. The model is then exported via Export to files in STL format and saved using a standardized nomenclature (e.g., CaseID_PatientInitials_State_Side.stl).

### 3.2. Part B: Mesh Optimization (MeshLab, Meshmixer and VMTK)

**Step 6: Purity Verification and Remeshing (MeshLab).** The raw STL is imported into MeshLab. To ensure that no micro-fragments survived the primary segmentation, Filters → Cleaning… → Remove Isolated Pieces is applied. To standardize the polygon distribution for downstream CFD meshing, isotropic remeshing is performed via Filters → Remeshing… → Isotropic Explicit Remeshing. The Target Length is set between 0.3 mm and 0.4 mm; a finer resolution of 0.3 mm is recommended for capturing complex fluid dynamics near the stent struts, whereas 0.4 mm is an acceptable compromise to reduce computational time in larger arterial segments. Between three and five iterations are applied. Adaptive remeshing is left unchecked, while Check Surface Distance is kept active with a Max Surface Distance ≤ 0.03.

**Step 7: Primary Smoothing (MeshLab).** The HC Laplacian Smooth filter is applied to relax the initial mesh tension, with the Smoothing steps set to 3. The model is then exported.

**Step 8: Localized Defect Removal (Autodesk Meshmixer).** The model is imported into Meshmixer. This stage requires manual intervention to eliminate non-physiological surface aberrations, and the brush tools are applied gently, analogous to smoothing soft clay. For spikes (narrow, sharp protrusions), the Select tool is used to highlight the defect base, Erase and Fill (F) is executed, and the area is blended using the Smooth MCV function. For bumps (broad, shallow protrusions), the RobustSmooth brush is used with Enable Refinement deactivated; the Strength is set to 15–20 and the Lazyness to 0 to gently depress the anomaly flush with the vessel wall. The corrected model is then exported.

**Step 9: Secondary and Final Surface Finishing (MeshLab).** The manually corrected model is re-imported into MeshLab, and a standard Laplacian Smooth filter (Filters → Smoothing… → Laplacian Smooth) is applied, executing two passes of 10 smoothing steps. Methodological Justification for Laplacian Smoothing: In standard soft-tissue anatomical modelling, Laplacian smoothing is often minimized or avoided due to its inherent tendency to cause volume shrinkage. However, the situation in a stented artery imaged by CTA is materially different. The attenuation of the metallic struts greatly exceeds that of contrast-enhanced blood, and the finite spatial resolution and reconstruction kernel of the scanner spread this attenuation into neighbouring voxels through partial-volume averaging and beam hardening. The resulting “blooming” artefact displaces the apparent stent boundary outward, typically by a fraction of a millimetre to more than a millimetre depending on strut thickness and material, tube voltage and reconstruction kernel [18,19,20]. Any threshold-based segmentation therefore returns a lumen–stent boundary that is systematically too large. This bias is not benign for the present application: for a given flow rate, wall shear stress varies inversely with the cube of the local radius, so even a modest overestimation of luminal calibre depresses computed wall shear stress appreciably and would bias the analysis towards spuriously low TAWSS and correspondingly elevated RRT—that is, towards precisely the findings the study seeks to test.

In this specific context the inward bias of the Laplacian operator is therefore not a defect but a corrective, acting in the direction opposite to that of the imaging artefact. Its use is deliberate and bounded. It is applied only after the mesh has been isotropically remeshed and manually cleaned, so that the displacement field is uniform and not driven by residual topological noise; it is applied in two controlled passes of ten steps rather than as a single aggressive pass, so that the intermediate geometry can be inspected; and it is followed immediately by Taubin smoothing, which arrests further contraction. Final Taubin Smoothing: Once the artificial stent volume has been appropriately reduced by the Laplacian filter, a final Taubin Smoothing is required (Filters → Smoothing… → Taubin Smooth). The parameters are configured to λ = 0.5 and μ = −0.53, and 20 to 30 iterations are applied. A standard default of 25 iterations is sufficient for most models; however, operators may increase this to 30 if visual inspection reveals persistent surface tension from severe “blooming” artefacts. Taubin smoothing is uniquely utilized at this final stage because it perfectly relaxes mesh tension and provides a high-fidelity surface polish while mathematically preventing any further volume loss. This essentially “locks in” the corrected dimensions, finalizing the watertight, hemodynamically accurate STL model for CFD simulations.

**Step 10: Surface mesh finalizing (VMTK—Vascular Modelling ToolKit).** The 3D surface mesh from Step 9 is loaded and read into the VMTK environment [12] (vmtksurfacereader -ifile) by providing the specific path and name of the previously created STL file. Once executed, this command parses the geometric data, points, and connectivity of the 3D model so that VMTK can process it; it is the first step in the VMTK pipeline (--pipe), which later passes the loaded surface dataset down the command pipeline as a VTP file (vmtksurfacereader -ofile). The 3D anatomical mesh is then smoothed (vmtksurfacesmoothing) using an algorithm that eliminates high-frequency surface artefacts while strictly minimizing volumetric shrinkage (Taubin Smoothing). The preferred choice for this study is a low value of 0.001 (-passband 0.001), in order to smooth only the finest, highest-frequency details while preserving the overall shape. The smoothing algorithm runs 30 times (-iterations 30) to produce a moderately noticeable smoothing effect without losing critical anatomical features. A surface truncation of the 3D geometry is then performed (vmtksurfaceclipper utility). This command provides an interactive graphical interface to precisely position clipping planes at the inlets and outlets. Opening these boundaries is a prerequisite for subsequent volumetric mesh generation intended for CFD analysis. The resulting modified surface mesh is then outputted (vmtksurfacewriter -ofile) in the desired format (usually an STL file), which is then imported into ANSA Cadense, a state-of-the-art CAE pre-processing software for complete model build-up, in order to generate the corresponding 3D mesh. Table 1 summarizes the workflow of VMTK in order to produce the STL file that will be imported in ANSA Cadense for mesh generation.

### 3.3. Part C: Volume Mesh and Blood Flow Simulation (ANSA Cadense, ANSYS Fluent)

**Figure 2:**
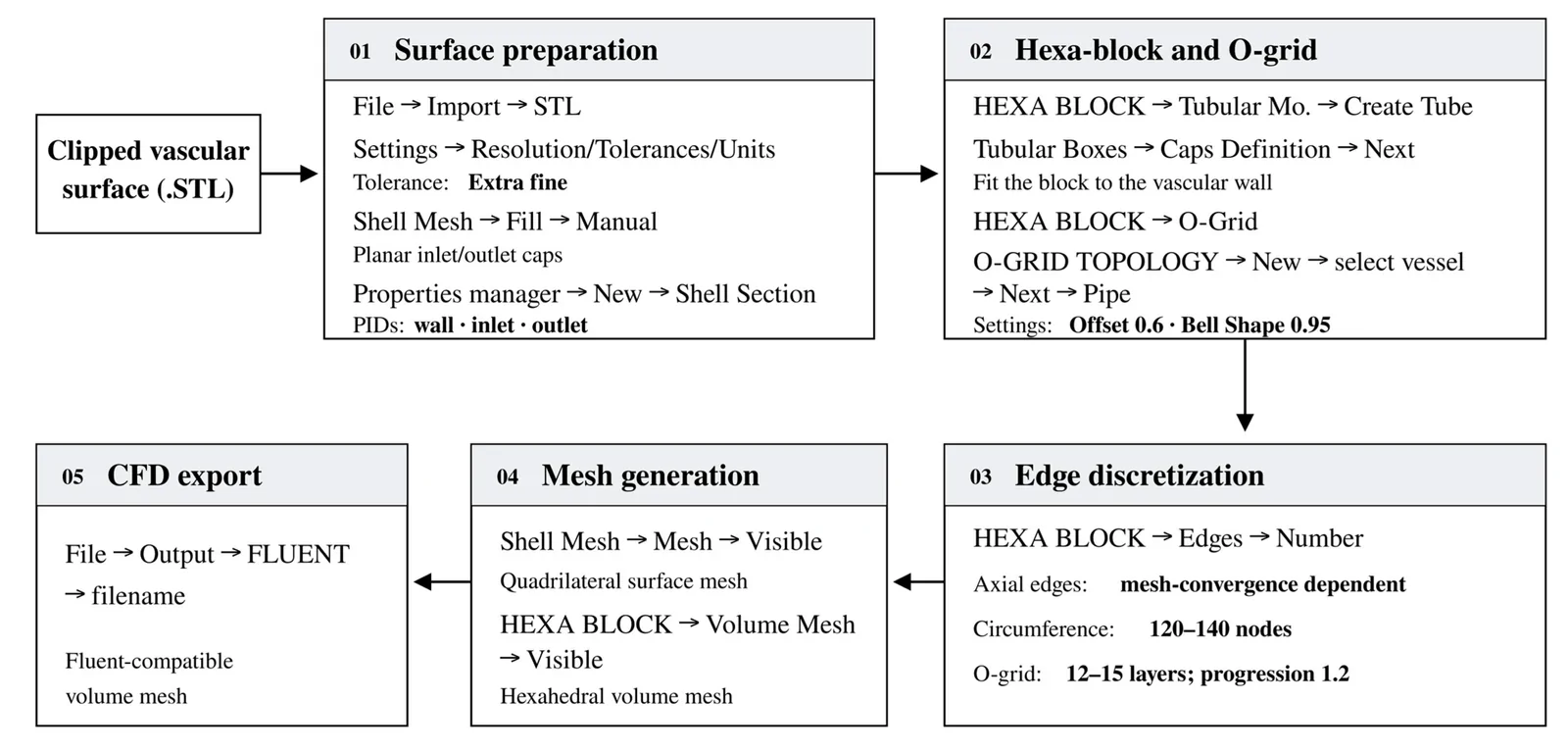
Workflow for generating a CFD-ready hexahedral vascular mesh in ANSA.

**Step 11: 3D mesh generation (ANSA Cadense).** During the first step the STL file is imported in ANSA [13] (File → Import → STL) and the Tolerance mode is set to extra fine under Settings → Resolution/Tolerances/Units. The clipped ends of the vessel are then filled with a planar surface using Shell Mesh → Fill → Manual on the mesh module. The three generated surfaces (wall, inlet, outlet) are then assigned an ID number that will be used during the CFD simulation. This is achieved through the Properties manager and by selecting New → Shell Section. The appropriate surface is selected and a specific PID (Property ID) is associated with each surface. The process is repeated for each surface and a separate ID is assigned to each one of them. Once the surface has been completed, the STL file of step 1 is loaded and a tube that engulfs the wall exactly is created following HEXA BLOCK → Tubular Mo. → Create Tube. The Tubular Boxes window opens up and by selecting Next in the Caps Definition the wall tube is formed. Inside the tube an area of small width is then created close to the wall to capture the boundary layer dynamics of the simulation. This is called an O-Grid and will be populated later with hexahedral elements of geometric progression for accurate results. It is formed by choosing HEXA BLOCK → O-Grid. The O-GRID TOPOLOGY windows opens up and press New. Highlight the whole vessel, press Next and insert the appropriate variables under Pipe. Usually, an Offset value of 0.6 and a Bell Shape pattern of 0.95 is sufficient in most cases. Once the O-Grid has been completed, the next step is the recreation of the surface mesh with high-quality elements such as quadrilateral elements. This is important in blood flow simulations because hexahedral elements (the three dimensional analog to quadrilateral elements) possess a series of advantages compared to prismatic elements, namely excellent flow alignment, low numerical diffusion, lower cell count for a specific level of accuracy, and a more precise calculation of the hemodynamic parameters (TAWSS, OSI, RRT, etc.) [15,21,22,23,24,25]. The number of nodes can then be determined under the HEXA BLOCK menu by selecting Edges → Number and by entering the desired number of nodes along the edges of the surface mesh. An additional step towards the completion of the surface mesh is the assignment of nodes along the circumference of the inlet/outlet and along the O-Grid. Mesh convergence studies have shown that a total number of 120 to 140 nodes along the circumference and 12 to 15 layers with a geometric progression of 1.2 for the O-Grid is sufficient in most of the cases. Both of these are assigned by following again the path Edges → Number and choosing the appropriate number of nodes. The generation of the produced mesh is then built using Shell Mesh → Mesh → Visible (assuming the whole mesh is shown on the screen). The volume mesh is then produced from HEXA BLOCK → Volume Mesh → Visible (assuming again the whole mesh is shown on the screen). As a final step, the volume mesh is exported as an STL file using File → Output → Fluent → filename. An appropriate workflow diagram summarizing the methodology of this section is shown in Figure 2.

**Figure 3:**
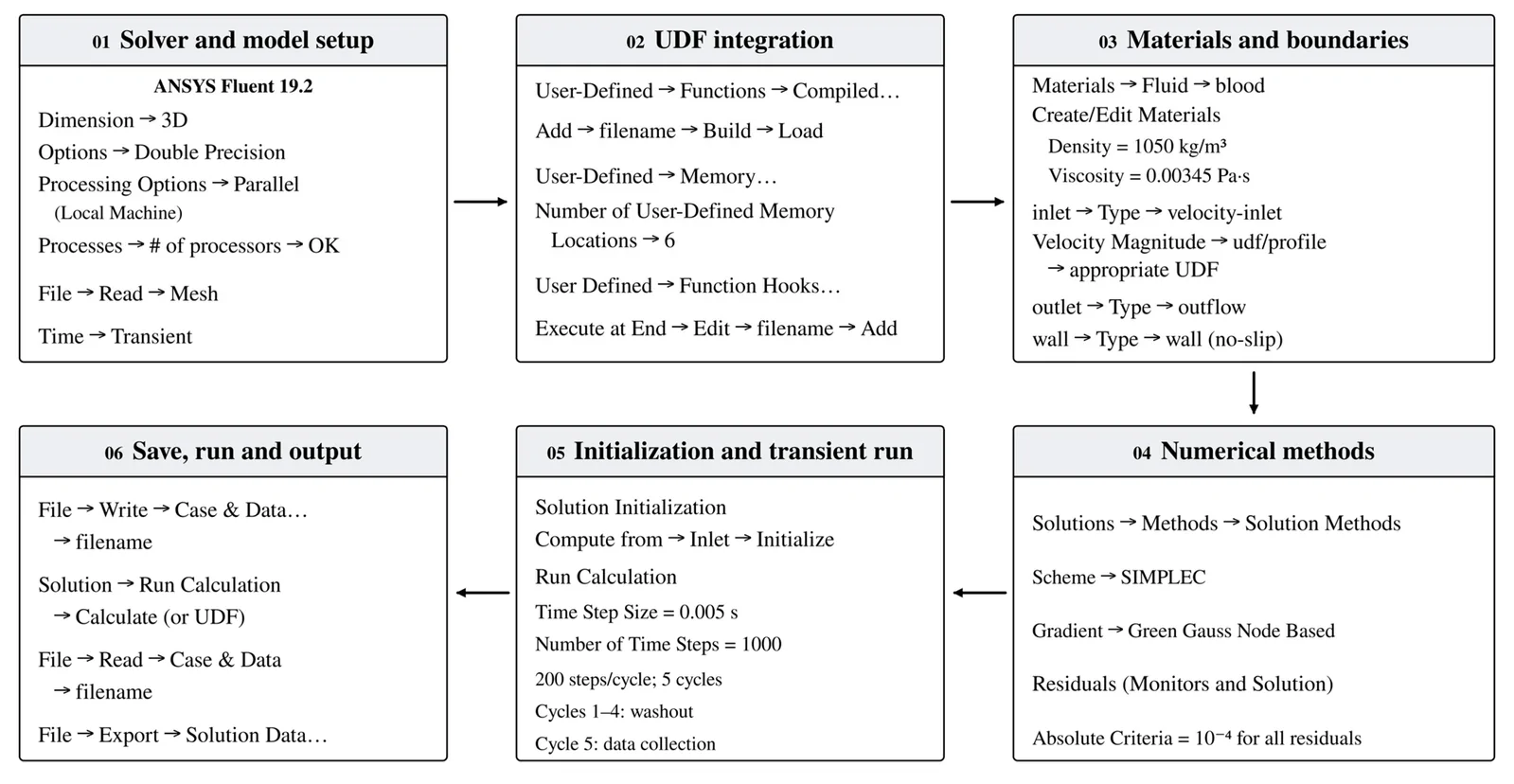
Workflow for CFD simulation using ANSYS Fluent.

**Step 12: CFD simulation (ANSYS Fluent).** Before the simulations, a mesh-convergence study was performed to ensure that the numerical results were independent of mesh resolution while maintaining a reasonable computational cost [23,25,26]. All simulations are performed using the Ansys Fluent 19.2 edition [24]. Set the initial parameters on Fluent Launcher as Dimension → 3D, Options → Double Precision, Processing Options → Parallel (Local Machine), and Processes → # of processors for parallel processing. Pressing OK launches the Fluent environment. Read the mesh (File → Read → Mesh) and assign a transient simulation with Time → Transient. Since the inlet velocity profile, the hemodynamic parameters to be calculated, and the rheological model are not by default incorporated in Fluent, User Defined Functions (UDF) must be defined. These are custom C-based programmes that can be compiled or dynamically linked to Fluent. They allow users to extend Fluent’s standard capabilities when built-in models cannot fully capture unique simulation physics, specific boundary conditions etc. For the needs of this study, the two UDFs for calculating the inlet velocity profile and the hemodynamic parameters are developed. These are compiled in the solver following User-Defined → Functions → Compiled… The Compiled UDFs window opens up. Pressing Add → filename → Build → Load loads each UDF into the solver. All the data that will be calculated during the simulation must be stored in specific locations for post-processing. This can be achieved with User-Defined → Memory… → Number of User-Defined Memory Locations → 6. In order to connect the custom C-written User-Defined Functions (UDFs) directly to the core solver, open the User-Defined Function Hooks dialogue box (User Defined → Function Hooks… → Execute at End → Edit → filename → Add). Define the physical properties of the fluid by choosing Materials → Fluid → air. Open the Create/Edit Materials and under Properties provide the density of blood (1050 kg/m^3^ for the needs of this study) and its viscosity (0.00345 Pa·s). Additionally, the user can also define the rheology of the simulated blood flow model by incorporating appropriate UDFs [23,24,27,28]. Set up the boundary conditions of the simulation (velocity at the inlet, pressure at the outlet and no-slip at the wall). Choose Boundary Conditions → inlet (right click) → Type → velocity-inlet. The Velocity Inlet window appears. From the Velocity Magnitude drop down menu under udf/profile select the appropriate UDF file. For pressure right click on outlet → Type → outflow. Finally, the no-slip boundary condition is set by following wall → Type → wall. Once the mesh, physical model, and boundary conditions have been defined, the set up moves to the Solution menu in order to manage the actual numerical computation. The Solution menu serves as the centralized, hierarchical framework for managing numerical execution within CFD workflows. Transitioning from the physics setup, the Solution menu organizes computational parameters into explicit, sequential sub-nodes. This structure allows researchers to rigorously define spatial discretization schemes, manipulate under-relaxation factors for numerical stability, and establish convergence criteria via residual and report definitions, ensuring reproducibility, precise convergence control, and streamlined execution of complex fluid simulations. To this end, the Pressure-Velocity Coupling window (under Solutions → Methods → Solution Methods) opens up. The set of parameters adopted for this study are given by Scheme → SIMPLEC and Gradient → Green Gauss Node Based. The Residual option (under Monitors and Solution) is then assigned a value which serves as the primary check and progress report for the simulation. Essentially, Residual measure the error in the conservation equations (like mass, momentum, and energy) from one iteration to the next and decides whether the time step will continue executing or will terminate. In order to ensure accurate results, the chosen value for all Absolute Criteria is set to 10^−4^. Once the set up has been completed, the initialization of the simulation needs to be performed which will establish the starting flow field by assigning the initial values (velocity and pressure) to every cell before iterating. As in all simulations, a realistic initial guess is crucial because it prevents mathematical divergence, ensures solver stability, and drastically reduces the time required to reach a converged solution. The initialization is done by choosing Solution Initialization → Compute from → Inlet → Initialize. The final step of the simulation is to define the appropriate step size and total number of time steps. Under Run Calculation the values of 0.005 (s) is set for the time step size and a total number of 1000 time steps is assumed. For a cardiac cycle of 1 s, the above set of parameters yield 200 time steps per cycle and five cycles (periods) in total. The first four periods ensure that all transient effects have been washed out before collecting the data during the last fifth cycle. The project is then saved following File → Write → Case & Data… → filename. The simulation can start either by Solution → Run Calculation → Calculate or using an appropriate UDF file if a more elaborate sequence is needed. When the simulation terminates, the outcome is accessed by File → Read → Case & Data → filename, or can be exported to a default format by File → Export → Solution Data… The principal steps described above are summarized schematically in Figure 3, providing a concise overview of the complete CFD simulation workflow implemented in ANSYS Fluent (Edition 19.2).

## 4. Expected Results

**Figure 4:**
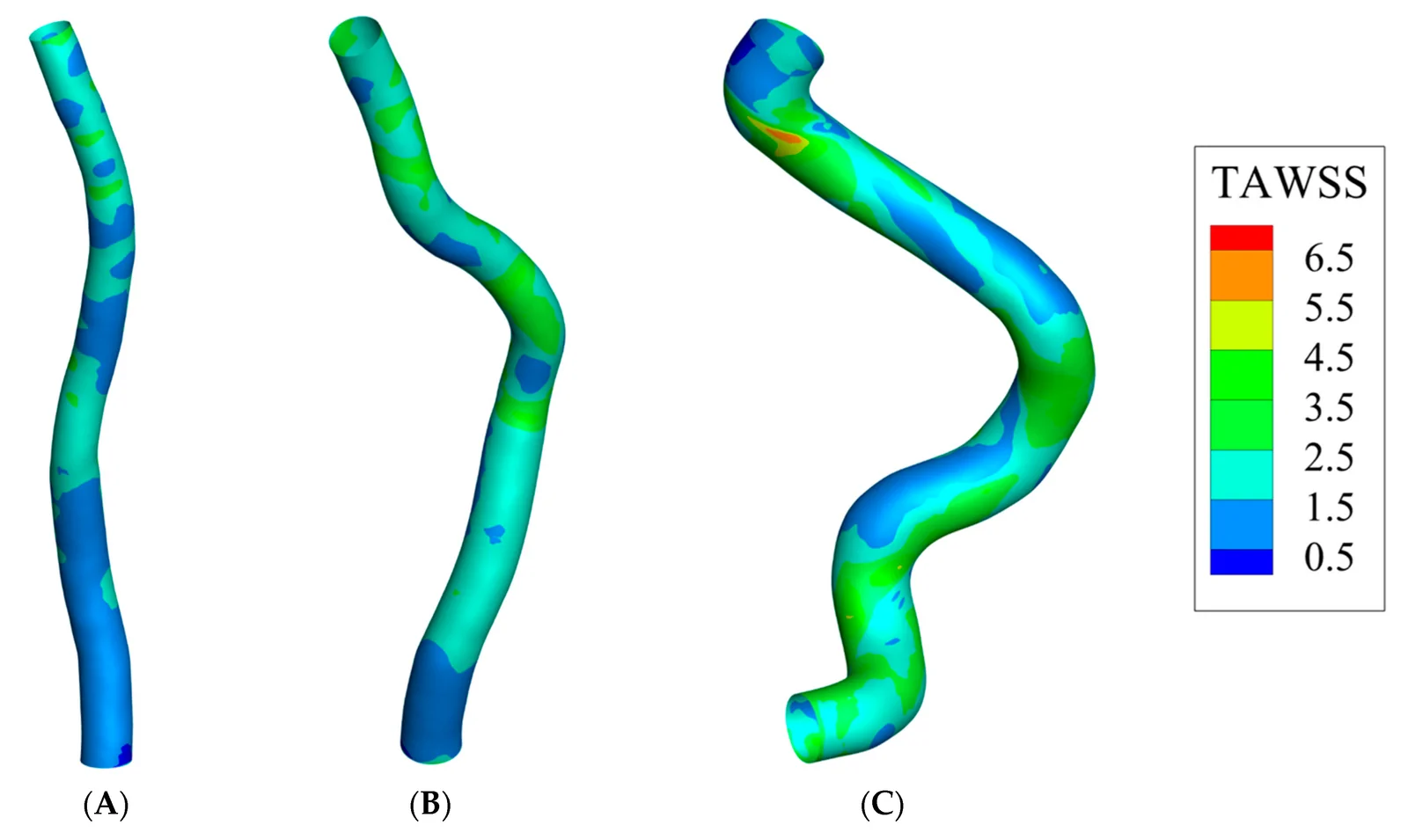
Representative patient-specific result for a popliteal stent-graft in three anatomical configurations, (**A**) Extension, (**B**) Mild flexion and (**C**) Extreme flexion. The surface maps show the time-averaged wall shear stress (TAWSS, Pa) computed for the same limb in each position. The change in the higher value recorded ranges from 3 Pa in extension to 6 Pa in extreme flexion.

The primary expected result of the overarching CFD study is to pinpoint the exact anatomical locations where hemodynamic conditions change significantly and negatively influence the vascular environment. In our study, the investigated stents are extensive, continuously spanning from above the knee joint to below it. A representative example is shown in Figure 4. Despite this complete scaffolding—which inherently limits acute localized mechanical kinking—clinical observations demonstrate that thrombosis still frequently occurs.

The overarching hypothesis is that dynamic knee flexion alters the global geometry of this long stented conduit, inducing complex internal hemodynamic disturbances even in segments that do not acutely bend. By running these highly accurate multi-positional models through a fluid solver, the analysis is expected to map complex localized flow variations. Specifically, we anticipate the CFD analysis will reveal critical alterations in the following key hemodynamic parameters:

- **Wall Shear Stress (WSS) and Time-Averaged Wall Shear Stress (TAWSS):** Identifying focal areas of abnormally low WSS, which are heavily correlated with endothelial dysfunction and localized thrombus formation.
- **Oscillatory Shear Index (OSI):** Mapping regions where the direction of blood flow fluctuates significantly during the cardiac cycle, indicating highly disturbed and pro-thrombotic flow regimes.
- **Relative Residence Time (RRT):** Highlighting areas of severe flow stagnation where blood particles remain trapped near the vessel wall, increasing the likelihood of coagulation.
- **Velocity Flow Profiles and Recirculation Zones:** Visualizing flow separation and the development of vortices at different angles of knee flexion.

To this end, the findings of the proposed protocol are divided into two categories. The primary endpoints, which include the assumed hemodynamic parameters (TAWSS, OSI, RRT), as well as the percentage of surface area under thrombogenic conditions [15,16,17,23], are separated from the secondary endpoints, such as the average values of these parameters at peak systole and late diastole. Figure 4 shows a preliminary result of a dramatic change in TAWSS by more than 100% between cases A (Extension) and C (Extreme flexion).

The correlation of these altered hemodynamic indices with the specific locations of clinically observed popliteal stent thrombosis is expected to provide definitive biomechanical evidence explaining why these devices fail in regions lacking severe mechanical bending. This standardized modelling approach ensures that the detected hemodynamic shifts are genuine physiological phenomena driven by positional changes, rather than artifactual errors stemming from poor initial mesh geometry.

## Figures and Tables

**Table 1:** VMTK commands used for vascular surface preprocessing.

Processing Step	VMTK Command(s)	Principal Options	Purpose
**Surface conversion**	vmtksurfacereader → vmtksurfacewriter	- ifile: .stl - ofile: .vtp	Imports the original STL surface and converts it to VTK PolyData format for subsequent processing.
**Surface smoothing**	vmtksurfacesmoothing	- passband 0.001 - iterations 30	Reduces surface irregularities and segmentation noise while preserving vascular geometry.
**Surface clipping**	vmtksurfaceclipper	- ifile: smoothed .vtp - ofile: clipped .vtp	Interactively removes unwanted portions of the model and defines the final inlet and outlet boundaries.
**Surface subdivision**	vmtksurfacesubdivision	Subdivision settings	Refines the polygonal surface by subdividing its elements before final export.
**Final surface export**	vmtksurfacereader → vmtksurfacewriter	- ifile: processed .vtp - ofile: .stl	Converts the processed VTP surface back to STL format for downstream meshing or simulation.
**Visual inspection**	vmtksurfaceviewer	Used after processing operation via --pipe	Provides visual quality control of the surface throughout the preprocessing workflow.

## Data Availability

The raw data supporting the conclusions of this article will be made available by the authors on request.
